# Evaluation of the EQ-5D-5L, EQ-VAS stand-alone component and Oxford knee score in the Australian knee arthroplasty population utilising minimally important difference, concurrent validity, predictive validity and responsiveness

**DOI:** 10.1186/s12955-023-02126-w

**Published:** 2023-05-10

**Authors:** D-Yin Lin, Tim Soon Cheok, Billingsley Kaambwa, Anthony J. Samson, Craig Morrison, Teik Chan, Hidde M. Kroon, Ruurd L. Jaarsma

**Affiliations:** 1 Department of Anesthesiology, Flinders Medical Centre, Adelaide, South Australia Australia; 2 College of Medicine and Public Health, Flinders University, Adelaide, South Australia Australia; 3 Department of Orthopaedic and Trauma Surgery, Flinders Medical Centre, Adelaide, South Australia Australia; 4 Department of Surgery, Royal Adelaide Hospital, Adelaide, South Australia Australia; 5 Faculty of Health and Medical Sciences, University of Adelaide, Adelaide, South Australia Australia

## Abstract

**Purpose:**

To evaluate the Oxford Knee Score (OKS), EQ-5D-5L utility index and EQ-5D visual analogue scale (EQ-VAS) for health-related quality of life outcome measurement in patients undergoing elective total knee arthroplasty (TKA) surgery.

**Methods:**

In this prospective multi-centre study, the OKS and EQ-5D-5L index scores were collected preoperatively, six weeks (6w) and six months (6 m) following TKA. The OKS, EQ-VAS and EQ-5D-5L index were evaluated for minimally important difference (MID), concurrent validity, predictive validity (Spearman's Rho of predicted and observed values from a generalised linear regression model (GLM)), responsiveness (effect size (ES) and standard response mean (SRM)). The MID for the individual patient was determined utilising two approaches; distribution-based and anchor-based.

**Results:**

533 patients were analysed. The EQ-5D-5L utility index showed good concurrent validity with the OKS (*r* = 0.72 preoperatively, 0.65 at 6w and 0.69 at 6 m). Predictive validity for the EQ-5D-5L index was lower than OKS when regressed. Responsiveness was large for all fields at 6w for the EQ-5D-5L and OKS (EQ-5D-5L ES 0.87, SRM 0.84; OKS ES 1.35, SRM 1.05) and 6 m (EQ-5D-5L index ES 1.31, SRM 0.95; OKS ES 1.69, SRM 1.59). The EQ-VAS returned poorer results, at 6w an ES of 0.37 (small) and SRM of 0.36 (small). At 6 m, the EQ-VAS had an ES of 0.59 (moderate) and SRM of 0.47 (small). It, however, had similar predictive validity to the OKS, and better than the EQ-5D-5L index. MID determined using anchor approach, was shown that for OKS at 6 weeks it was 8.84 ± 9.28 and at 6 months 13.37 ± 9.89. For the EQ-5D-5L index at 6 weeks MID was 0.23 ± 0.39, and at 6 months 0.26 ± 0.36.

**Conclusions:**

The EQ-5D-5L index score and the OKS demonstrate good concurrent validity. The EQ-5D-5L index demonstrated lower predictive validity at 6w, and 6 m than the OKS, and both PROMs had adequate responsiveness. The EQ-VAS had poorer responsiveness but better predictive validity than the EQ-5D-5L index.

This article includes MID estimates for the Australian knee arthroplasty population.

**Supplementary Information:**

The online version contains supplementary material available at 10.1186/s12955-023-02126-w.

## Introduction

Total knee arthroplasty (TKA) is a safe and cost-effective surgery for patients with osteoarthritis who do not respond to medical therapy alone [1] and in Australia, a total of 54,102 replacements were performed per year from 2017 – 2018 (218 per 100,000). [2] Despite the well-established safety data and patient improvements published over the last 20 years [1], the measurement of patient-related outcomes, including functional change or improvement, are not as clear-cut for TKA compared to other orthopaedic surgery such as total hip arthroplasty. [3, 4].

Patient-reported outcome measures (PROMS) are used as a measurement tool to evaluate patient and health economic outcomes, with an example being the 5-level version of the EuroQol 5 Dimensions (EQ-5D-5L index score). This standardized health-related quality of life (HRQoL) questionnaire was initially developed in 1990 as a 3-level version designed to assess general health for five dimensions. [5, 6] In 2011, it was revised to a 5-level version (EQ-5D-5L index) with five levels and five dimensions to reduce granularity in health response and reduce the ceiling effect. [7] The EQ-5D questionnaires are some of the most widely used PROMs globally; in some countries, such as the United Kingdom, it is used to calculate quality adjusted life years used in cost-utility analysis [8–10].

While extensively used in other parts of the world, the EQ-5D-5L index score has not yet been well validated for the Australian orthopaedic population for HRQoL assessment. [11] The results of the EQ-5D-5L index score PROM are converted into vectors which are five-digit codes representing a health state. For example, 11,111 is full health, and 55,555 represents the worst health. There are 3,125 possible health states. These are mapped onto a single utility index using a country-specific value set. To date, more than 25 countries have validated country-specific EQ-5D-5L value sets for various patient populations. [12].

The EQ-VAS is a stand-alone component of the EQ-5D-5L index, in which a patient self-reports their impression of their general health and functionality. Compared with the in-depth, question-and-answer format of the ED-5D-5L index, the EQ-VAS is seen as a simpler and less ambiguous format. [13] The Oxford Knee Score (OKS) is a validated PROM specifically developed to assess function and pain in patients undergoing TKA. [14] It had been utilised to assess the concurrent validity of the EQ-5D-5L index in TKA patients in other countries. [15].

The minimally important difference is defined as the smallest PROM score change, which is perceived significantly by patients or clinicians. [16] The MID is 'anchored' by using a satisfaction survey to identify patients who experienced a change in their functional status considered perceptible and clinically important. Changes in functional status were measured using a five-point Likert scale at one year postoperatively scored as either (1) "very satisfied", (2) "satisfied" (3) "neither satisfied nor dissatisfied", (4) "dissatisfied", or (5) "very dissatisfied". Patients whose functional change was 4 or 2 were considered to have experienced some change equivalent to the MID. [17] It is generally considered that the anchor-based approach is the optimal method for evaluation of MID as it yields a direct expression of the patient’s preferences and values. [16] The distribution-based method of MID estimation assesses the distribution of scores around the mean of the measurement of interest, for example standard deviation. [18].

Concurrent validity describes the extent of the method being tested to assess an outcome correlates with an established method to measure the same. Here the EQ-5D-5L index will be tested against the established OKS. Predictive validity describes the association between baseline and follow-up outcomes which is highly valued in this cohort, as it has implications for surgical suitability for individual patients. Responsiveness, a measure of the sensitivity of PROMs to reflect a change in health status over time, is also tested.

### Outcome measure

This study aims to compare the EQ-5D-5L utility index and EQ-VAS against the OKS in Australian patients undergoing total knee arthroplasty using the minimally important difference (MID), concurrent and predictive validity.

### Patients and methods

This multi-centre prospective trial was conducted at two large tertiary teaching hospitals in Adelaide, Australia. A group of orthopaedic surgeons operate routinely at both sites, performing approximately 300 knee arthroplasty surgeries annually. However, the number of patients operated on in 2020 was reduced to approximately 150 due to SARS Covid-19-related restrictions. The local governing Human Research Ethics Committee granted multi-centre approval (SALHN/329.17).

All consecutive adult patients undergoing elective total knee arthroplasty surgery were prospectively enrolled over a nearly three-year period from 8^th^ January 2018 to 1^st^ of October 2020, with a six-month follow-up until 2^nd^ April 2021. Indication for surgery was predominantly osteoarthritis, all joint replacements were primary operations only. Informed consent was obtained from all participants, and baseline demographics were recorded for all patients, including age, gender, body mass index (BMI) and the Charlson comorbidity index (CCI) [19, 20].

Data were recorded at three different time points (preoperatively, six weeks and six months postoperatively) by one dedicated research assistant, using scripted questionnaires via telephone or a written survey sent by postal mail. At all three time points, two validated PROMs were used: the Oxford Knee Score (OKS) [21] and the EQ-5D-5L index score [5] including the EQ-VAS stand-alone component. Data were keyed into a password-secured database and stored on the hospital computer network.

Patients were included for analysis if they had complete quality of life data. This was defined as completing the EQ-5D-5L index score and OKS for the three time points.

### Oxford knee score

The OKS is a joint-specific PROM [22, 23] which has been extensively utilised over the last 20 years. It assesses six fields (pain, walking, physical activity, function, quality of life and psychological wellbeing), with each field containing 2 questions, making up a total of 12 questions. Each question is scored on a 5-point discrete visual analogue scale where higher scores indicate better function. The final score is a sum tally of the individual question scores, with a range of 0 to 48. The OKS has previously been utilised as a comparator for responsiveness with PROMs such as the EQ-5D-3L and SF-12 in a similar patient population, albeit in different countries than Australia. [24, 25].

### EQ-5D-5L index and EQ-VAS

The EuroQol Group designed the EQ-5D-5L index to quantify general health in adults. Using a 5-point scale (none, slight, moderate, severe and extreme/unable to perform), it evaluates the fields of mobility, self-care, usual activities, anxiety/depression and pain/discomfort. Based on the general Australian population, preference weights can be attached to each of the EQ-5D-5L health states. These were determined through a discrete choice experiment approach [26]. Utility indices vary from − 0.676 to 1, with higher utilities signifying a better HRQoL.

The EQ-VAS is a vertical visual analogue scale which constitutes a part of the EQ-5D-5L index score and can also be used as a stand-alone component. Patients are to rate their general health from 0 to 100, with higher numeric scores denoting a better function. The EQ-5D-5L index questionnaire is established on specific national value sets or the generic Western Preference Pattern. [27] It has been validated in approximately 28 countries as of 2022 [28–31].

### Statistical analysis

All statistical analyses were performed utilising STATA version 17 (StataCorp, Texas, USA). Continuous variables (age, BMI, CCI) were expressed as means and standard deviations. The categorical variable (gender) was expressed as percentages (counts). A *p*-value of < 0.05 was considered statistically significant.

### Concurrent validity, predictive validity and agreement

For analysis of concurrent validity, Spearman's correlation coefficient (rho, ρ) was utilised to compare the EQ-5D-5L index and EQ-VAS against the OKS. The strength of the relationship can be assessed as low/weak (*ρ* < 0.25), fair (*ρ* = 0.25 to < 0.50), good (*ρ* = 0.50–0.75), or excellent (*ρ* > 0.75). This magnitude of rank order correlations was sourced from previous publications on the same area. [32, 33].

Predictive validity was ascertained using a regression framework, whilst controlling for confounders. We utilised generalized linear models with the 6-week and 6-month postoperative PROMs as the dependent variable, and the preoperative values and baseline characteristics as independent variables. Depending on the distribution of the dependant variable, the most appropriate distribution family and canonical link function were chosen. Multiple families (including the Gaussian, inverse Gaussian, Poisson, and Gamma distributions) were trialled when there was difficulty ascertaining the appropriate family of distribution. The best fitting model was then selected based on low Akaike's Information Criteria and Bayesian Information Criteria scores. The average marginal effect with respect to preoperative score was used to compare models if different distribution families were utilised.

The agreement between the EQ-5D-5L index and the OKS was measured using Bland–Altman analysis at all three measurement points.

### Responsiveness

Responsiveness is a measure of the sensitivity of PROMs to reflect the change in health status over time. For this study, we compared measurements at baseline, 6 weeks and 6 months follow-up using paired t-tests. Further assessment of responsiveness was quantified using effect size (ES) and standardized response mean (SRM).

The effect size was calculated using the formula: effect size equals the mean difference from baseline divided by the standard deviation at baseline.

The standard response mean was calculated using the formula: standard response mean equals mean difference from baseline divided by the standard deviation of difference.

ES and SRM were classified according to Cohen’s rule of thumb, as large (≥ 0.8), moderate (0.5–0.79) or small (< 0.5). [34] Both ES and SRM are standardized measures of change over time in health, independent of sample size.

### Influence of baseline characteristics on PROMs

Regression analysis of the baseline characteristics (age, gender, BMI and CCI) was performed using generalised linear models with the preoperative EQ-5D-5L index, EQ-VAS and OKS as independent variables. The preoperative PROMs were used as the dependant variables, and depending on the distribution, an appropriate distribution family and canonical link function were chosen using the same approach taking in the predictive validity analysis. The coefficient, standard error and p-values were recorded.

### Determination of minimally important difference

Minimally important difference (MID) is defined as the smallest change in score, which is perceived as important by patients or clinicians. [35] The MID for the cohorts was defined as the change in PROM score for patients who responded as satisfied [2] or dissatisfied [4] to the anchor question at one year. The MID was determined using two approaches: distribution-based approach, and the anchor-based approach.

The distribution-based approach defined MID as half the baseline standard deviation of the PROM scores [36] For both the anchor-based approach, we quantified satisfaction based on the anchor question (satisfaction rating). We then calculated Spearman's correlation coefficient to assess the correlation between the measured score and the satisfaction rating. The MID calculation would not be performed if the correlation coefficient was less than 0.25. While calculating the MID using the anchor-based approach, we considered a satisfaction score of 2 or 4 as having experienced some MID-equivalent change. The MID was then taken as the mean changes in scores of the patients who scored 2 or 4.

## Results

In total, the database had 797 patients, of which 96 were excluded as they did not have a preoperative questionnaire completed, 115 did not have any postoperative questionnaires answered, and a further 9 had their operation cancelled. There were statistically insignificant differences in characteristics between those with complete data and those with missing data for nearly all demographic characteristics. Out of 12 comparisons, only 2 statistically significant differences were seen with another borderline significant (Additional file 1: Appendix 1). Therefore, complete case analysis was conducted.

Six hundred seventy-three knee arthroplasty patients with preoperative and postoperative questionnaires completed were identified from the database. Of these, 140 had preoperative and 6w data, and the further 533 had complete data for preoperative, 6w and also 6 months. All 673 with both pre- and postoperative data were included in the study. The mean age of our cohort at the time of surgery was 68.3 ± 9.6 years old, and 59.14% (398/673) were female. The mean preoperative BMI was 31.9 ± 5.7 and the mean CCI was 72.0 ± 22.4%. A summary of baseline characteristics can be found in Table 1. Early complications of arthroplasty recorded at 6 weeks included 20 cases of venous thromboembolism, 19 cases of additional antibiotic use, eight cases of peri-prosthetic fractures, seven cases of myocardial infarctions, five cases of cerebrovascular events, four cases of postoperative stiffness limiting rehabilitation and two cases of periprosthetic infections requiring re-operation. Eleven patients had more than one complication, and 610 patients of the total 673 included reported no complications. Of the 533 patients who were followed up until 6 months, 53 of them had early complications.

**Table 1:** Baseline Characteristics

Age (mean ± SD)	68.3 ± 9.6
Gender (M/F)	275/398
BMI (mean ± SD)	31.9 ± 5.6
CCI (mean ± SD)	72.0 ± 22.4

*SD* Standard Difference, *M/F* Male/Female, *BMI* Body Mass Index

**Fig.1:**
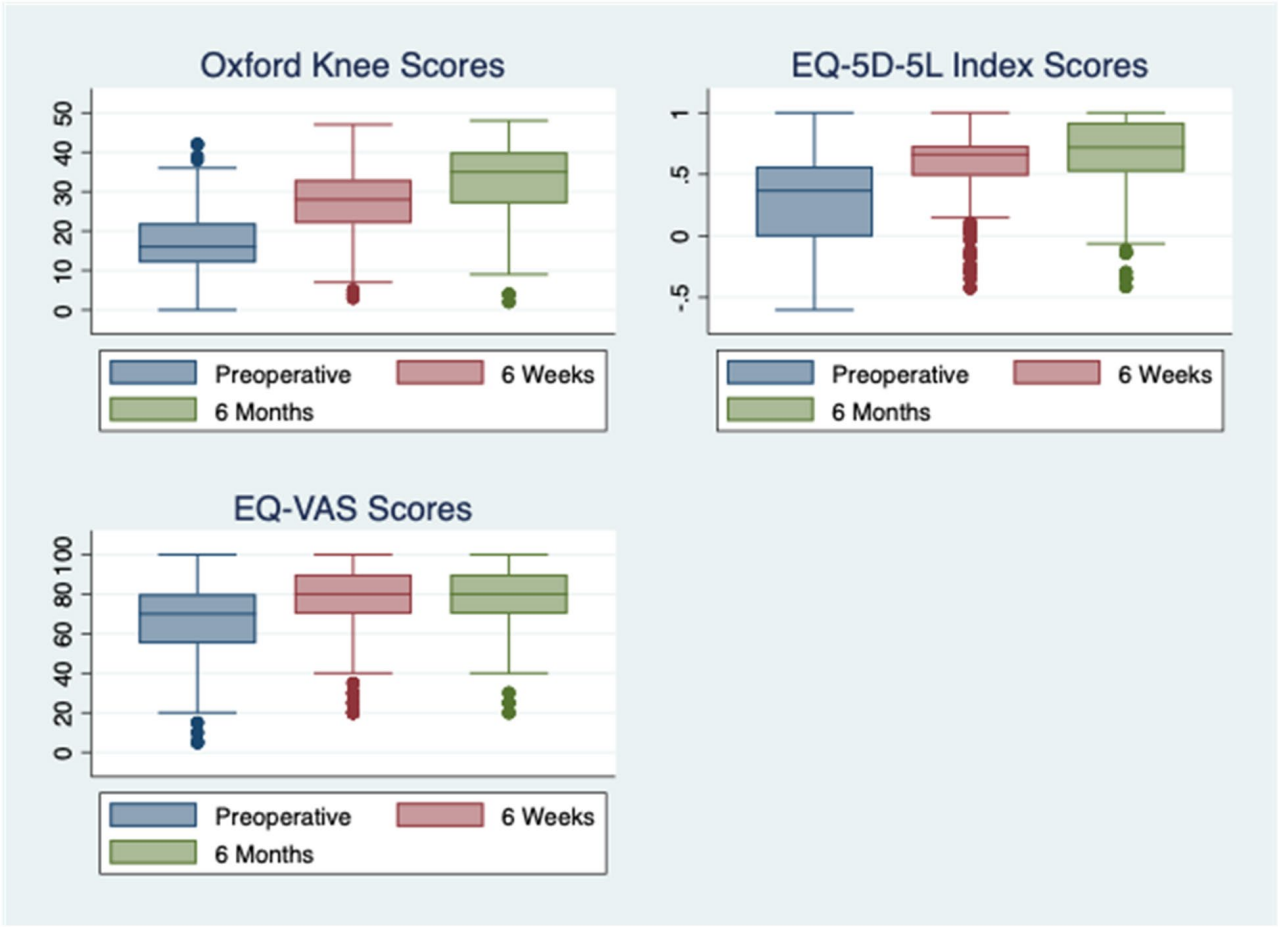
Boxplots Showing Distribution of PROMs Scores over Time

Boxplots for the distributions of scores at baseline (preoperative), 6 weeks and 6 months are shown in Fig. 1.

Number of patient responses to the satisfaction survey at one year were as follows:

- 1 (Very satisfied): 196 (48.2%)
- 2 (Satisfied): 114 (28%)
- 3 (Neither Satisfied Nor Dissatisfied): 62 (15.2%)
- 4 (Dissatisfied): 24 (5.9%)
- 5 (Very Dissatisfied): 11 (2.7%)

A summary of baseline characteristics can be found in Table 1.

### Concurrent validity, predictive validity and agreement

EQ-5D-5L index showed good concurrent validity when compared to OKS at baseline, 6 weeks, and 6 months postoperative, with a Spearman's coefficient of 0.72, 0.65 and 0.69, respectively. EQ-VAS had fair concurrent validity when compared to OKS at baseline, 6 weeks, and 6 months postoperative, with a Spearman's coefficient of 0.31, 0.46 and 0.49 respectively (Table 2).

**Table 2:** Concurrent and Predictive Validity

Concurrent Validity (Spearman’s Coefficients)				
	EQ-5D-5L		EQ-VAS	
Preoperative	0.72 (Good)		0.31 (Fair)	
6 Weeks	0.65 (Good)		0.46 (Fair)	
6 Months	0.69 (Good)		0.49 (Fair)	
**Predictive Validity**				
	6 Weeks		6 Months	
	Average Marginal Effect (Standard Error)	Model (Link)	Average Marginal Effect (Standard Error)	Model (Link)
OKS	0.33 (0.05)	Gamma (Negative Inverse)	0.37 (0.06)	Gamma (Negative Inverse)
EQ-5D-5L index	0.25 (0.03)	Gaussian (Identity)	0.23 (0.04)	Gaussian (Identity)
EQ-VAS	0.34 (0.04)	Gamma (Negative Inverse)	0.31 (0.04)	Gamma (Negative Inverse)

*OKS* Oxford Knee Score

Predictive validity for each of the three different PROMs score was determined using generalized linear models, with regression to baseline scores and covariates. In all cases, the distribution that provided the best model fit was the Gamma distribution with a canonical negative inverse link. The average marginal effects for the preoperative score were recorded and displayed in Table 2. The EQ-5D-5L index score showed lower predictive validity when compared to OKS at 6 weeks and 6 months. EQ-VAS, however, showed similar predictive validity compared to OKS at 6 weeks and 6 months.

**Fig. 2:**
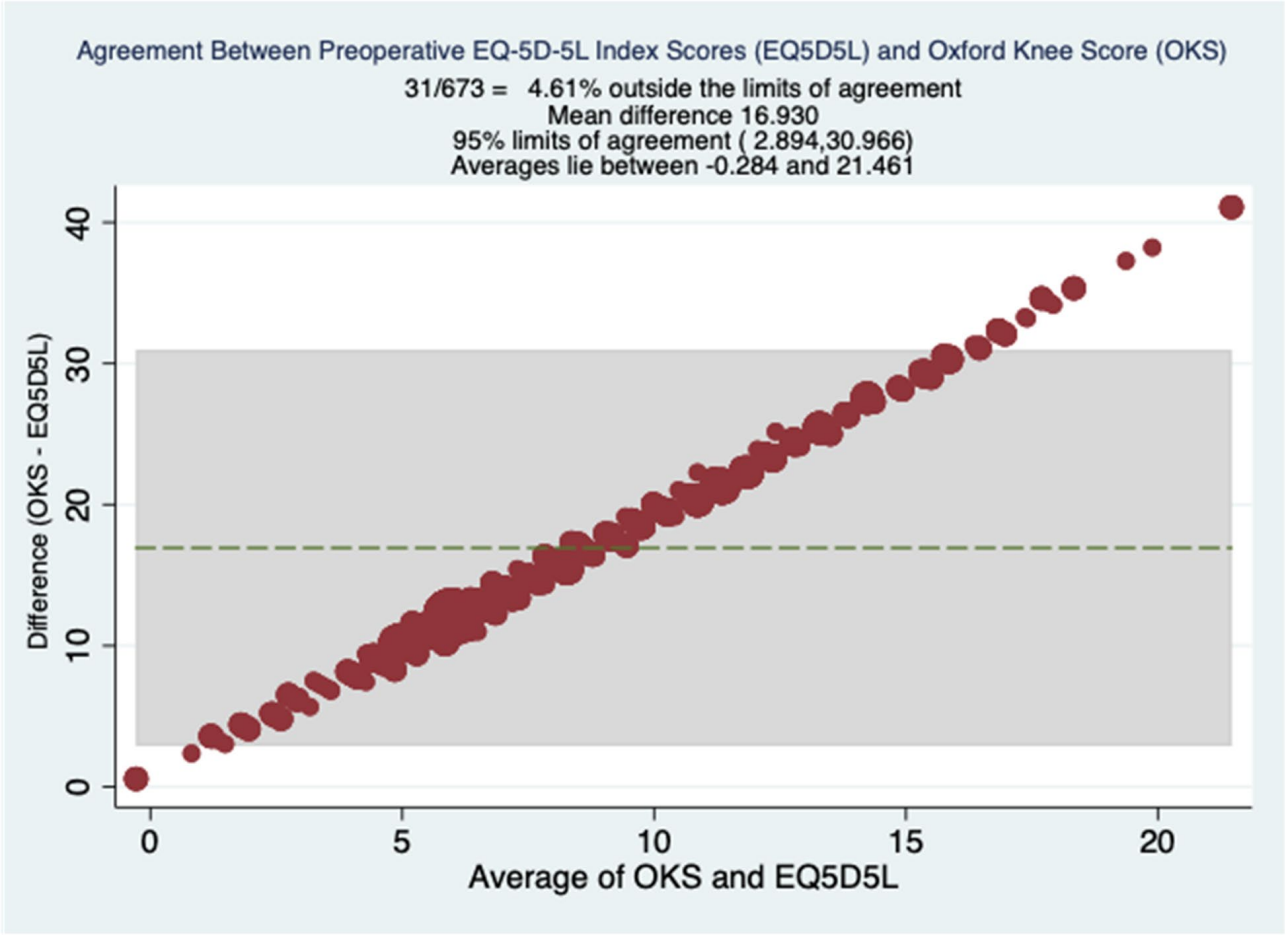
Preoperative Bland Altman Plots

**Fig. 3:**
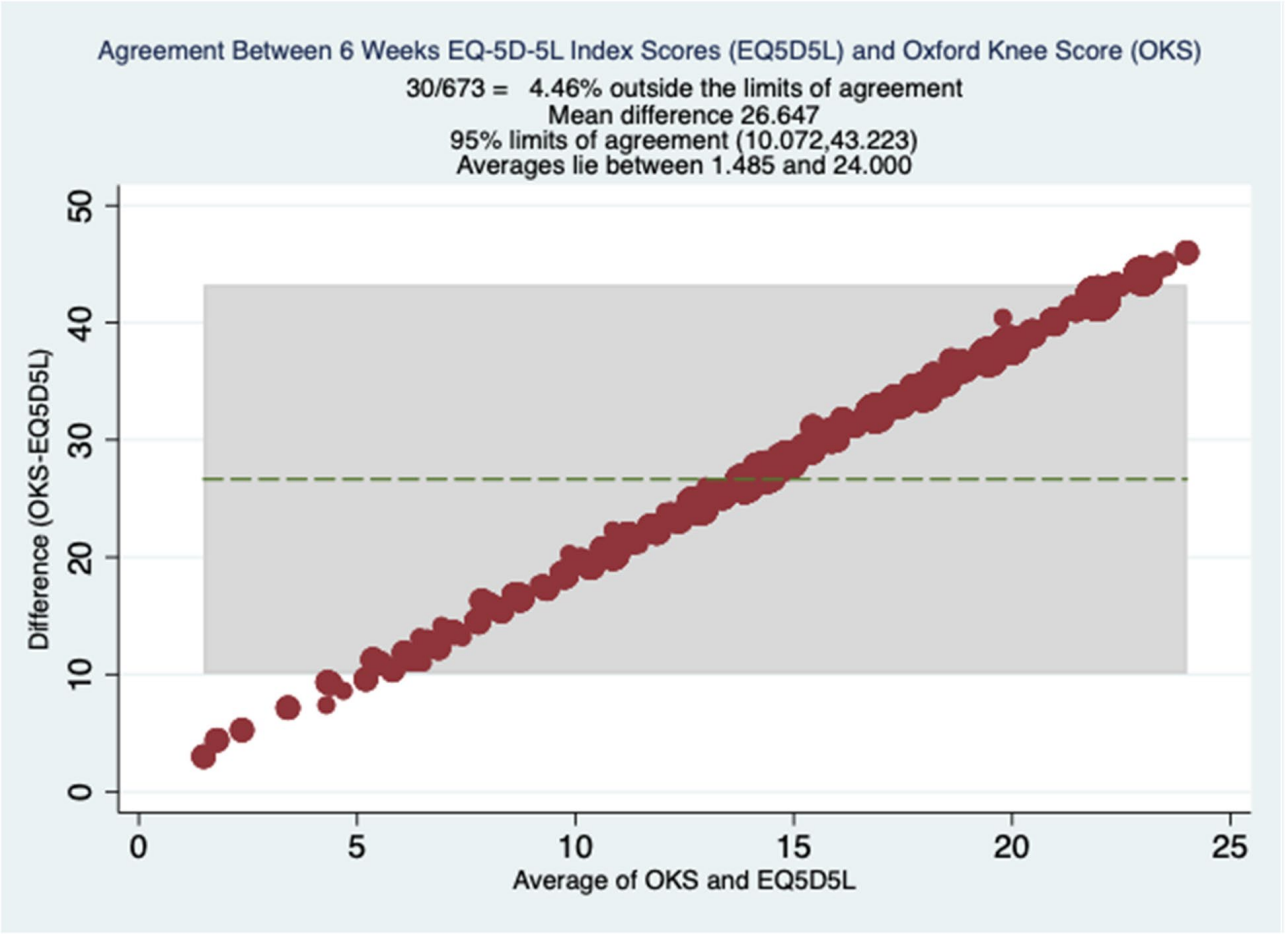
Bland Altman Plots at 6 Weeks

**Fig. 4:**
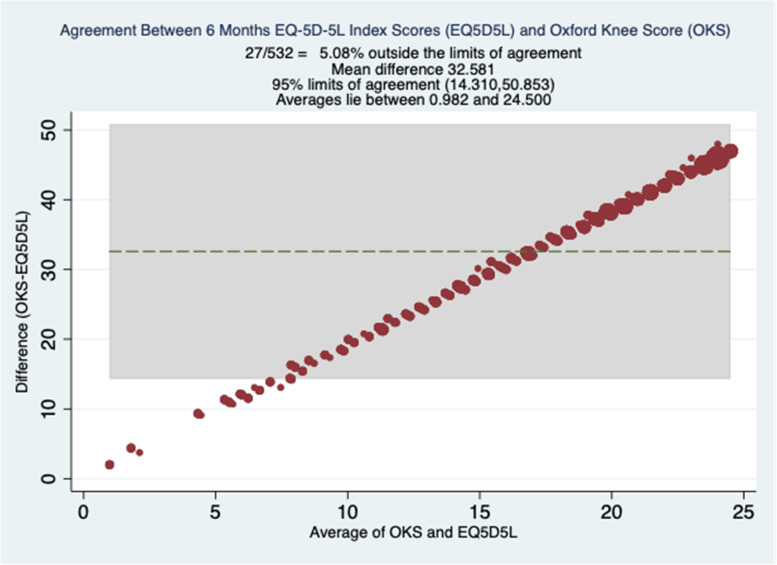
Bland Altman Plots at 6 Months

Bland Altman's plot showed good agreement between OKS and EQ-5D-5L index at preoperative, 6 weeks and 6 months, with approximately 95% of data points within the limits of agreement. These plots are shown in Figs. 2, 3 and 4.

### Responsiveness

At 6 weeks, all three PROMs showed significant differences between baseline and follow-up scores. Both OKS and EQ-5D-5L index had a large ES and SRM, although the actual estimate for OKS was larger. The ES for OKS and EQ-5D-5L index was 1.35 and 0.87, respectively, and the SRM was 1.05 and 0.84, respectively. The EQ-VAS had a small ES and SRM of 0.37 and 0.36, respectively.

At 6 months, all three PROMs again showed a significant difference between baseline and follow-up scores: The ES for OKS, EQ-5D-5L index, and EQ-VAS were 1.69, 1.31 and 0.59, respectively, and the SRM was 1.59, 0.95 and 0.47 respectively. These findings are detailed in Table 3.

**Table 3:** Responsiveness of PROMs

(a) 6 Weeks						
	Preoperative	6 Weeks	Mean Difference	Paired t-Test	Effect Size	Standard Response Mean
OKS	17.23 ± 7.41	27.25 ± 8.62	10.02 ± 9.58	< 0.0001	1.35 (Large)	1.05 (Large)
EQ-5D-5L index score	0.30 ± 0.35	0.61 ± 0.26	0.31 ± 0.37	< 0.0001	0.87 (Large)	0.84 (Large)
EQ-VAS	67.71 ± 19.07	74.79 ± 16.54	7.08 ± 19.76	< 0.0001	0.37 (Small)	0.36 (Small)
(b) 6 Months						
	Preoperative	6 Months	Mean Difference	Paired t-Test	Effect Size	Standard Response Mean
OKS	17.20 ± 7.33	33.26 ± 9.52	16.07 ± 10.08	< 0.0001	1.69 (Large)	1.59 (Large)
EQ-5D-5L index score	0.30 ± 0.35	0.67 ± 0.28	0.36 ± 0.38	< 0.0001	1.31 (Large)	0.95 (Large)
EQ-VAS	67.81 ± 19.14	77.05 ± 15.62	9.24 ± 19.68	< 0.0001	0.59 (Moderate)	0.47 (Small)

### Influence of baseline characteristics on PROMs

Since EQ-5D-5L scores had negative values, it was determined that the Gaussian family of distribution with a canonical identity link was most appropriate compared to both OKS and EQ-VAS, which had non-negative distributions. Therefore, the Gamma distribution provided the best fit and was hence used for the final model. All three preoperative PROMs were significantly affected by CCI. EQ-VAS was additionally significantly affected by BMI (Table 4).

**Table 4:** Regression Analysis with respect to Baseline Characteristics using Preoperative PROMs as the Dependant Variables

	Oxford Knee Score			EQ-5D-5L Index Score			EQ-VAS Score		
	Coefficient	SE	p-Value	Coefficient	SE	p-Value	Coefficient	SE	p-Value
Age	1.00	0.00	0.305	1.00	0.00	0.343	1.00	0.00	0.112
Gender – Male	1.00	0.00	0.100	1.01	0.03	0.816	1.00	0.00	0.129
BMI	1.00	0.00	0.862	1.00	0.00	0.384	1.00	0.00	0.014*
CCI	0.98	0.01	0.004*	1.23	0.10	0.011*	1.00	0.00	0.001*

*BMI* Body Mass Index, *CCI* Charlson Comorbidity Index

### Minimally important difference

As measured using the distribution-based method, the MID for OKS and EQ-5D-5L index were 3.70 and 0.18, respectively. When the anchor-based technique was utilised, the MID for OKS at 6 weeks and 6 months was 8.84 ± 9.28 and 13.37 ± 9.89, respectively. The MID for the EQ-5D-5L index scores were 0.23 ± 0.39 and 0.26 ± 0.36 at 6 weeks and 6 months, respectively (Table 5).

**Table 5:** Minimum Important Difference (MID)

	Spearman’s Correlation	Distribution Technique (0.5*Baseline SD)	Anchor Technique
OKS at 6 Weeks	0.34	3.70	8.84 ± 9.28
OKS at 6 Months	0.53		13.37 ± 9.89
EQ-5D-5L index at 6 Weeks	0.34	0.18	0.23 ± 0.39
EQ-5D-5L index at 6 Months	0.50		0.26 ± 0.36

*OKS* Oxford Knee Score

## Discussion

This analysis is an empirical validation of the EQ-5D-5L index’s suitability in assessing HRQoL amongst knee arthroplasty patients using experienced-based patient data from a prospective multi-centre study database, with the correlation between the Oxford Knee Scores, EQ-VAS, and the EQ-5D-5L index PROMs. The findings support the utilization of the EQ-5D-5L index as a valid and reliable instrument in assessing HRQoL amongst these patients, but it must be noted that the OKS outperformed the EQ-5D-5L index in all fields. The EQ-VAS had poorer responsiveness than the EQ-5D-5L index, but better predictive validity.

The EQ-VAS as a stand-alone measure showed a smaller ES than the EQ-5D-5L index at both six weeks (0.37 versus 0.87 respectively, *p* < 0.0001) and six months (0.59 versus 1.31 respectively, *p* < 0.0001). The SRM was large for the EQ-5D-5L index score at the six-week and six-month time points, but only small for the EQ-VAS. However, the EQ-VAS had better predictive validity than the EQ-5D-5L index but comparable validity to the OKS. This suggests a higher predictive value for postoperative recovery and could be used as an adjunct to the EQ-5D-5L index score. An explanation for this may be the broader nature of the EQ-VAS (ie. not proscribed by the domains or items as in the OKS or EQ-5D-5L index descriptive system), which allows the patients to consider more quality of life constructs in their subjective rating of health. This is beneficial for patient stratification and counselling regarding realistic rehabilitation expectations and postsurgical results.

The EQ-VAS standalone component was only fair in terms of concurrent validity. The OKS is a joint-specific PROM, whereas the EQ-5D-5L index is designed to assess overall functionality. For example, someone who can compensate enough to perform daily tasks and cope well with the mental burden of an arthritic knee on the EQ-5D-5L index, may record gait disturbances and set specific difficulties with mobility on the OKS. We chose the OKS as a comparator for this validation as it is widely used and has significant items that overlap with the EQ-5D-5L index. For example, both feature mobility, pain/discomfort and usual activities. Hence, they should be utilised concurrently to complement each other, instead of being considered as substitutes for one another.

This study analysed MID via two approaches; anchor-based and distribution-based. An estimate of MID in this patient population is important clinically as it will indicate when a particular patient would notice a benefit from knee arthroplasty surgery. It is important in study design, as any new treatment being investigated should aim to detect a difference at least equal to the MID. Non-inferiority studies should aim to show that the difference between groups is less than the MID for the Australian orthopaedic population. [37].

The longitudinal nature of this study with multiple time points allows evaluation of the incremental changes in the population and the differences in the performance of both PROMs. The experience-based and prospective nature of this data is also a strong point.

Generalizability of this study is high, as surgical technique and perioperative management is consistent with standard practice in Australia, and worldwide.

The EQ-5D-5L index has been assessed against other PROMs in the TKA population in previous publications, and found to to be more responsive (ES and SRM) than other scores in reflecting health related changes in this group. [38] Conner-Spady et al. found a MID of 0.20 for TKA patients for the EQ-5D-5L index. [15] They reported a wide variation in the MID with the percentage agreement of responder classification using 2SEM versus MID ranging from 79.6 to 99.6% for the EQ-5D-5L and from 69.4 to 94.8% for the Oxford scores. Recommendations included utilising multiple PROMs for HRQoL assessment in future studies. Our study also found a wide variation, with a similar MID result to those found by the previous studies.

There is a paucity of literature for TKA and concurrent and predictive validity, but comparable literature for total hip arthroplasty in the Australian population has previously illustrated that the EQ-5D-5L index and the OHS demonstrate strong concurrent validity. The EQ-5D-5L index had similar predictive validity at 6w and 6 m. [11].

Some limitations of this study have to be addressed. There were approximately 21% missing data for patients at six months. Therefore, these patients had to be excluded, introducing a response bias.

Future research should include further validation of these clinically relevant PROMs, as well as perhaps corroboration of the baseline MID for knee arthroplasty patients in Australia.

## Conclusions

In conclusion, The EQ-5D-5L index and the Oxford Knee Score demonstrate good concurrent validity in this study. EQ-5D-5L index revealed a large effect size at six weeks and six months postoperatively, but smaller than the OKS at all time points. Both PROMs had adequate responsiveness. However, the OKS outperformed the EQ-5D-5L in all fields. The EQ-VAS had poorer responsiveness than the EQ-5D-5L index, but better predictive validity when used as a stand-alone component.

The EQ-5D-5L index PROM is suitable to quantify general health-related quality of life in the Australian knee arthroplasty patient population. Still, given the OKS superior performance in terms of predictive validity and responsiveness, it should be favoured for use above the EQ-5D-5L. Ideally, both can be used to complement each other with an assessment of a joint specific PROM in OKS and a more generalised health assessment in EQ-5D-5L.

This article establishes a baseline MID for the Australian knee arthroplasty patient population, which can be incorporated into further research or utilised for patient counselling in the perioperative phase.

## Supplementary Information

**Additional file 1.**

## Data Availability

Data available upon request.

## Abbreviations

TKA: Total knee arthroplasty
6w: Six weeks
6 m: Six months
ES: Effect size
SRM: Standardized response mean
PROMs: Patient reported outcome measures
OKS: Oxford Knee Score
VAS: Visual Analogue Scale
CCI: Charlson Comorbidity Index
HRQoL: Health Related Quality of Life
MID: Minimally Important Difference
TMLE: Targeted Maximum Likelihood Estimation
BMI: Body mass index
TTO: Time Trade-Off
