# Supplementary material for: Evaluation of the EQ-5D-5L, EQ-VAS stand-alone component and Oxford knee score in the Australian knee arthroplasty population utilising minimally important difference, concurrent validity, predictive validity and responsiveness
Source: Health Qual Life Outcomes. 2023 May 10;21:41. doi: 10.1186/s12955-023-02126-w (PMC10170024; doi:10.1186/s12955-023-02126-w)
Supplement: Supplementary file 1 — Additional file 1. [file 12955_2023_2126_MOESM1_ESM.docx]

Appendix 1.

There were statistically insignificant differences in characteristics between those with complete data and those with missing data for nearly all demographic characteristics. Out of 12 comparisons, only 2 statistically significant differences were seen with another borderline. Crucially, there were statistically significant differences in the PROM scores between the two groups (please see results below).

**Sorted by Complications at 6W**

**Baseline Demographics**

Age: No Difference on t test (p=0.118)

1. No complications: 68.09 +/- 9.64
2. Complications: 70.07 +/- 9.03

Gender (M/F): No Difference on Chi2 (p=0.639)

1. No Complications: 251/359
2. Complications: 24/39

BMI: No difference on t test (p=0.374)

1. No Complications: 31.98 +/- 5.81
2. Complications: 31.28 +/- 5.03

CCI: **Those with complications had a significantly lower CCI** (p=0.004)

1. No Complications: 72.76 +/- 21.68
2. Complications: 64.13 +/- 27.64

**PROMS Baseline**

OKS: No significant difference on t test (p=0.055)

1. No Complications: 17.41 +/-7.39
2. Complications: 15.52 +/-7.41

EQ5D5L (Index): No significant difference on t test (p=0.556)

1. No Complications: 0.30 +/- 0.35
2. Complications: 0.33 +/- 0.37

**Sorted by Complications at Presence of Follow Up at 6 Months**

**Baseline Demographics**

Age: No Difference on t test (p=0.066)

1. No FU: 66.95 +/- 9.38
2. FU: 68.63 +/- 9.63

Gender (M/F): **Greater male dominance in the FU group on Chi2 (p=0.049)**

1. No FU: 47/93
2. FU: 228/305

BMI: No difference on t test (p=0.328)

1. No FU: 32.35 +/- 6.42
2. Complications: 31.81 +/- 5.55

CCI: **Those with FU had a significantly lower CCI** (p=0.043)

1. No FU: 75.49 +/- 21.58
2. FU: 71.06 +/- 22.56

**PROMS Baseline**

OKS: No significant difference on t test (p=0.820)

1. No FU: 17.36 +/-7.72
2. FU: 17.20 +/-7.33

EQ5D5L (Index): No significant difference on t test (p=0.710)

1. No FU: 0.29 +/- 0.35
2. FU: 0.30 +/- 0.35

Given the above, we felt that assuming that data were missing completely at random was appropriate as common methods of dealing with missing data (such as mean and multiple imputation) can lead to over or underestimation of MIDs in an instance such as ours. (1)

REFERENCES

1. Woaye-Hune P, Hardouin JB, Lehur PA, Meurette G, Vanier A. Practical issues encountered while determining Minimal Clinically Important Difference in Patient-Reported Outcomes. Health Qual Life Outcomes. 2020;18(1):156.
